# Does patient self-management education of primary care professionals improve patient outcomes: a systematic review

**DOI:** 10.1186/s12875-018-0847-x

**Published:** 2018-09-29

**Authors:** Andree Rochfort, Sinead Beirne, Gillian Doran, Patricia Patton, Jochen Gensichen, Ilkka Kunnamo, Susan Smith, Tina Eriksson, Claire Collins

**Affiliations:** 1 Irish College of General Practitioners, 4-5 Lincoln Place, Dublin 2, Ireland; 2 Institute of General Practice and Family Medicine, University Hospital, LMU, Munich, Germany; 3 University of Helsinki, EBM Guidelines, Duodecim Medical Publications Ltd, Helsinki, Finland; 4 Royal College of Surgeons in Ireland, Dublin, Ireland; 5 GP Copenhagen, Copenhagen, Denmark

**Keywords:** Self-management, Patient empowerment, Primary care, Chronic conditions

## Abstract

**Background:**

Patient self-management support is recognised as a key component of chronic care. Education and training for health professionals has been shown in the literature to be associated with better uptake, implementation and effectiveness of self-management programs, however, there is no clear evidence regarding whether this training results in improved health outcomes for patients with chronic conditions.

**Methods:**

A systematic review was undertaken using the PRISMA guidelines using the Cochrane Library, PubMEd, ERIC, EMBASE, CINAHL, PsycINFO, Web searches, Hand searches and Bibliographies. Articles published from inception to September 1st, 2013 were included. Systematic reviews, Meta-analysis, Randomized controlled trials (RCTs), Controlled clinical trials, Interrupted time series and Controlled before and after studies, which reported on primary care health professionals’ continuing education or evidence-based medicine/education on patient self-management for any chronic condition, were included. A minimum of two reviewers participated independently at each stage of review.

**Results:**

From 7533 abstracts found, only two papers provided evidence on the effectiveness of self-management education for primary healthcare professionals in terms of measured outcomes in patients. These two articles show improvement in patient outcomes for chronic back pain and diabetes based on RCTs. The educational interventions with health professionals spanned a range of techniques and modalities but both RCTs included a motivational interviewing component.

**Conclusions:**

Before and up to 2 years after the incorporation of patient empowerment for self-management into the WONCA Europe definition of general practice, there was a scarcity of high quality evidence showing improved outcomes for patients as a result of educating health professionals in patient self-management of chronic conditions.

**Electronic supplementary material:**

The online version of this article (10.1186/s12875-018-0847-x) contains supplementary material, which is available to authorized users.

## Key messages

Despite a vast literature on the topic of patient self-management, evidence on the association between training of health professionals in patient self-management with measured patient health outcomes was rare prior to and up to 2 years after its incorporation into the WONCA Europe definition of general practice.

However, the limited available evidence suggests that specific training programmes for primary care health professionals (a) may improve and support patient competencies for self-management and (b) may improve quality of life for patients with chronic conditions.

## Background

The European Definition of General Practice by the World Organization of National Colleges, Academies and Academic Associations of General Practitioners/Family Physicians (WONCA) in the European Region, lists 12 characteristics within six core competencies to define the activities of general practice / family medicine (GP/FM) [1]. The twelfth characteristic of general practice “*Promotion of patient empowerment and self-management*” was officially approved in 2011 [1]. It follows therefore, that European family physicians agree that among the various disciplines of medicine, GP/FM has a key role in harnessing patient autonomy to develop their expertise in managing their own health and wellness [2].

Promoting patient self-management (PSM) in the European definition of General Practice is listed under the core competency of “Patient-centered care”, in keeping with scientific evidence on patient empowerment within general practice [3]. The concept of patient empowerment for patient self-management in chronic conditions has been further explored by the European Society for Quality and Safety in General Practice (EQuiP) [4].

The World Health Organisation (WHO) defines chronic conditions as those that encompass disability and disease that people ‘live with’ for extended periods of time [5]. They include non-communicable diseases (NCD), such as cardiovascular diseases, cancers, respiratory diseases and diabetes, and are associated with lifestyle factors and patient behaviours and account for 70% of deaths globally (40 million deaths) in 2017 [6]. By 2030, the total annual number of NCD deaths is projected to increase to 52 million per year [6]. This escalation of the prevalence of chronic conditions is a significant factor in the increasingly heavy workload in family medicine internationally, which is also increasing in complexity as people live longer often with multiple co-existing chronic conditions.

By definition, chronic disease is not reversible or curable [6]. The Chronic Care Model (CCM) [7] is an internationally accepted model for the management of NCD and specifies self-management support as a key component. Patient self-management can be described as *“a set of tasks and processes that are used by a patient to maintain wellness in the presence of an ongoing illness* [8] *and it may also encompass prevention...”* [9]. In addition to knowledge of the disease and treatment options, patient self-management *“involves active involvement in decision making, coping with signs and symptoms of disease, making lifestyle changes and managing the impact of the disease on life”* [10].

The benefits of supporting patients to implement and maintain self-management skills have been shown to improve patients’ self-care and more appropriate utilisation of health services [11–13]. Improved patient self-management can also reduce health care costs through fewer outpatient visits [14–17] and fewer hospital admissions [18–20]. Lifestyle interventions by patients have been shown to have clinical benefit in a wide range of conditions such as diabetes, coronary heart disease, heart failure, and rheumatoid arthritis [21].

In the Chronic Care Model, achieving optimum outcomes for patients requires a productive interaction between “an informed activated patient and a prepared proactive practice team” [22]. Many studies focus on the role of *patient education* in self-management, yet no systematic reviews are published on whether *clinician education* to improve patient self-management has an effect on patient outcomes. A preliminary literature review [23] indicated that effective patient self-management support would require specific training of primary care health professionals. Education and training have been identified as a potential way of engaging primary care clinicians in patient self-management support [24]. However, though existing studies suggest that health professional training is associated with better uptake and implementation of patient self-management programs [9, 14, 25, 26], it is not clear which type of professional training this might involve, or whether it actually improved patient outcomes.

The primary aim of this systematic review was to examine the effectiveness of educational interventions for primary care professionals that are designed to improve their support for patient self-management of chronic conditions and improve patient outcomes. The timeframe was specifically chosen in order to establish if this evidence was available prior to and up to 2 years after the concept of patient empowerment for self-management was introduced into the WONCA Europe definition of general practice. Furthermore, this systematic review was used to inform the subsequent project work packages, which included the creation of an online educational module and its evaluation.

## Methods

A systematic review was undertaken using the PRISMA guidelines [27] and follows the methodology outlined in the PROSPERO registered protocol (Database registration number: CRD42013004418) [28].

### Sourcing information

Two specialist subject librarians assisted in the development of the search strategy designed to identify internationally recognised terminology in peer-reviewed journals. Full details of this strategy are available in the published protocol [28]. Six databases were searched - Cochrane Library, PubMed, ERIC, EMBASE, CINAHL and PsycINFO - in addition to Web searches, Hand searches and Bibliographies. Articles published in advance of September 1st, 2013 were included in the review, with the search conducted by GD and PP. The full search terms and sample search are shown in ‘Additional file 1’. The timeframe was deliberately chosen in order to coincide with the inclusion of the concept of patient empowerment in WONCA Europe’s definition of general practice. It was also the first work package of a larger project. It is intended to repeat the systematic review in 2018.

### Selection criteria

Studies with the following designs were included: systematic reviews, randomized controlled trials (RCTs), controlled clinical trials, interrupted time series, and controlled before and after studies.

Participants were physicians in primary care settings, other clinicians in primary care settings and patients 18+ years with chronic conditions in primary care settings. Included interventions had an educational focus designed to train primary care clinicians to support patient self-management. This review was concerned with all chronic conditions as they occur generically in the primary care setting, rather than focusing on any one specific chronic condition. Only articles including reference to patient outcomes, measured using validated measurement scales, were included. The primary patient outcome was change in patients’ self-management behaviours; the secondary outcomes were changes in physical health measures, health behaviours including medical adherence and compliance, service utilisation, psychological health, psycho-social function (e.g. Quality of Life, SF36, SF12) physical functioning and knowledge.

The eligibility of studies was determined using the inclusion and exclusion criteria listed in the registered proposal and shown in Table 1.

**Table 1:** Inclusion and exclusion criteria

Inclusion Criteria	Exclusion Criteria	Exclusion code
English papers	Non- English papers	Eng
Adults (18+)	Study population < 18	Age
Primary Care/Community	Secondary Care/Hospital	Not PC
Chronic conditions, chronic illness, chronic disease, non-communicable disease (NCD)	Acute conditions	Acute
Study Type- Systematic reviews, meta- analysis, RCTs, controlled clinical trials, interrupted time series. Controlled before and after studies	Study Type- Qualitative studies, populations studies, surveys, cross sectional, uncontrolled before and after studies (cohort)	Study
Education and training of primary care Health Professionals for patient education in promoting change, behaviour change, lifestyle change, patient engagement, patient empowerment, motivational skills, patient collaboration, patient adherence and compliance, Patient self-management, decision making, patient problem- solving	Not education/training of health care professionals	Int
	Not primary care health professionals	Pop
	Primary outcome measures not included	Out
	Direct patient education only	Edu
Continuing education / CME / Lifelong learning / Evidence based medicine	Guideline adherence, clinical performance	Guid
All studies published to September 2013	Organisational interventions	Org
	Financial changes and incentives	Fi
	Regulatory interventions	Reg

### Data extraction

All abstracts were reviewed using the RefWorks package to categorise the abstracts identified by the search. The initial review of abstracts was undertaken by SB with 10% of same re-checked by AR. The full text articles of all those considered to be of possible relevance to the systematic review were read independently by SB, JG and CC and categorised using the same exclusion reasons. Disagreements were reviewed by AR. The final list of full text articles were then reviewed by JG to confirm relevance. The quality assessment and extraction of thematic content of the final list of articles applicable to the systematic review question were considered by CC and AR.

### Quality assessment

We assessed risk of bias and overall quality of individual studies using the Quality of Assessment Tool for Quantitative Studies [29] (Tables 2, 3 and 4). For each study, reviewers rated six components (selection bias, study design, confounders, blinding, data collection methods, and withdrawals and dropouts) leading to an overall methodological quality rating for each study of strong, moderate, or weak, with strong quality indicating a low risk of bias. Reviewers resolved rating disagreements through discussion.

**Table 2:** Risk of bias

Bias	Becker et al. [30]	Rubak et al. [31]
Random sequence generation (selection bias)	Low - Selection by central permuted block randomisation	Low - Selection by drawing lots
Allocation Concealment (selection bias)	Low	Unclear-Insufficient information provided
Blinding of participants and personnel (performance bias)	High – Blinding of participants and personnel was not possible	High – Blinding of participants and personnel was not possible
Blinding of outcome assessment (detection bias)	High – Self-reported outcomes	High – Self-reported outcomes
Incomplete outcome data (attrition bias)	Low – Clear participant flow reported	Low – Clear participant flow reported
Selective reporting (reporting bias)	Low – The published report includes all expected outcomes	Low – The published report includes all expected outcomes
Other bias	Unclear – but unlikely. Insufficient information to assess whether another important risk of bias exists	Unclear – but possible; no baseline data. Insufficient information to assess whether another important risk of bias exists

**Table 3:** Quality assessment using EPHPP tool

Component	Becker et al. [30]	Rubak et al. [31]
Selection Bias 1. Are the individuals selected to participate likely to be representative of the target populations?	Can’t tell = 4	Can’t tell = 4
Selection Bias 2. What percentage of the selected individuals agreed to participate?	Less than 60% agreement = 3	Can’t tell = 5
SELECTION BIAS RATING	WEAK	WEAK
Study design	Randomized control trial = 1	Randomized control trial = 1
Was the study described as randomized?	Yes	Yes
Was the method of randomization described?	Yes	Yes
Was the randomization process appropriate?	Yes	Yes
Study design rating	Strong	Strong
Were there important differences between groups prior to the intervention?	No = 2	No = 2
What percentage of relevant confounders were controlled?	N/A	N/A
Confounders rating	Strong	Strong
Were the outcome assessors aware of the intervention status of participants?	Can’t tell = 3	Can’t tell = 3
Were the participants aware of the research question?	No = 2	No = 2
Blinding rating	Moderate	Moderate
Were data collection tools shown to be valid?	Yes = 1	Yes = 1
Were data collections tools shown to be reliable?	Yes = 1	Yes = 1
Data collection rating	Strong	Strong
Were withdrawals and drop-outs reported in terms of numbers/reasons?	Yes = 1	Yes = 1
Percentage of participants completing the study	80 = − 100% = 1	80 = −100% = 1
Withdrawals and drop outs rating	Strong	Strong
Intervention Integrity: What percentage of participants received the allocated intervention?	80 = −100% = 1	80 = − 100% = 1
Was the consistency of the intervention measured	Can’t tell = 3	Can’t tell = 3
Is it likely that subjects received an unintended intervention that may influence results?	No = 5	No = 5
Analyses: Unit of allocation	Practice	Practice
Unit of analysis	Individual	Individual
Are the statistical methods appropriate for the study design?	Yes = 1	Yes = 1
Is the analysis performed by intervention allocation status (ITT) rather than actual intervention received?	Yes = 1	Yes = 1

**Table 4:** Summary of Global rating for Quality using EPHPP Quality Assessment tool

Component	Becker et al. [30]	Rubak et al. [31]
Selection Bias	Weak	Weak
Study Design	Strong	Strong
Confounders	Strong	Strong
Blinding	Moderate	Moderate
Data Collection Methods	Strong	Strong
Withdrawals and Dropouts	Strong	Strong
Global rating	Moderate	Moderate

Criteria for global rating; 1. Strong = no weak ratings 2. Moderate = one weak rating, 3. Weak = two or more weak ratings

### Data synthesis

We performed a narrative data synthesis as the clinical heterogeneity and differences in outcomes in the two studies meant meta-analysis would have been inappropriate.

## Results

### Study review and selection

**Fig. 1:**
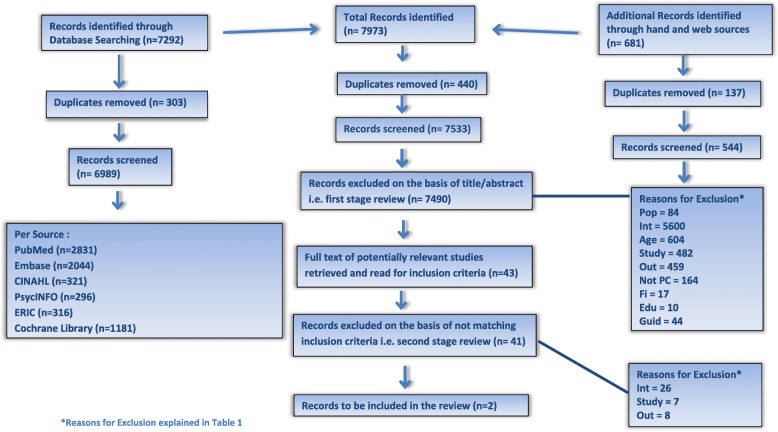
Summary of Systematic Review Process

Overall 7533 abstracts were reviewed following removal of duplicates from the database and hand/web searches; of these, 43 full text articles were retrieved and read (Fig. 1). Following this second stage review, only two articles that reported patient outcomes were included in the systematic review.

The two papers were both RCTs of educational interventions in primary care health professionals and examined their impact on patient outcome measures (Table 5).

**Table 5:** Study Description

Author Country Year	Design/Intervention	Analysis (unit of analysis/power calculation)	Objective measurement/Follow-up period	Successful Educational Aspects	Limitations
Becker et al. Germany 2008 [30]	Cluster RCT with 2 intervention arms and 1 control arm. Control arm guidelines only (C). Two intervention groups - both received a multifaceted general practitioner education and GI one additionally received motivational counselling training for practice nurses (MC) GI and MC trained in using LBP guideline for the DEGAM – 4 modules: 3 interaction seminars; information given on local facilities; 2 individual educational visits by study nurses. In MC group, 2 nurses per practice received 2 full day workshops and 1–3 supervision sessions and study coordinators contacted the nurses regularly.	Unit of analysis is the patient. Power calculation for small effects. Drop out analyses included.	Main Outcome: Hannover Functional Ability Questionnaire for Measuring Back Pain Related Functional Limitations. Secondary outcomes: Freiburg Questionnaire on Physical Ability; Korffs severity of chronic pain scale; Euro Quality of life questionnaire; Fear Avoidance Beliefs questionnaire; Days of sick leave. Follow-up: 6 and 12 months.	After 6 months: functional capacity improvement more pronounced in intervention groups and significantly so for adjusted differed between MC and C groups; both GI and MC patients significantly less days in pain during previous 6 months and less patients in intervention groups indicated suffering permanent pain than C patients. 12 months: more pronounced reduction in days in pain in GI and MC compared to C group. Patients in MC group showed significant improvement in quality of life. Clinical guidelines improve outcome; physician education has a little benefit, motivational interviewing adds slightly more benefit but probably only useful for some patients.	Inclusion rate 44% which might be due to selection bias. Patient sample had wide representation of pain qualities and quantities as well as different motivational stages for behaviour change, so individual differences in effects of interventions may be masked. Included patients may have had lower levels of pain, higher physical activity and readiness for change than general LBP patients in general – may reduce external validity of the study. Validity of the FQPA for a primary care sample with low disability may be insufficient and may limit its discriminative power. Insufficient counselling sessions to draw conclusions.
Rubak et al. Denmark 2009 [31]	One year follow up of an RCT/1.5 day residential MI course for GPs and a half day follow up twice during first year. Both I and C groups had half day course on intensive treatment of type 2 diabetes.	Unit of analysis is the patient. Sample size determined by power analysis.	Health Care Climates Questionnaire; Treatment Self-regulation Questionnaire; Diabetes Illness Representation Questionnaire; Summary of Diabetes self-care activities. Follow-up: 12 months.	Patients in I group significantly more autonomous in their choice of action towards behavioural changes and more motivated to change behaviours; also significantly more aware of the importance of controlling their diabetes for specific factors.	Not blinded at randomization. No baseline data; Patients were newly diagnosed so there was no change behaviour and no statements regarding diabetes at baseline. No blinding of behavioural changes –Hawthorne effect may exist; but if so, existed in both groups. Involvement in study may have influenced and diminished effect of MI.

The primary outcome of this review is the effectiveness of educational interventions in terms of patient outcomes. Our results show that education and skills training of primary care health professionals may improve patient performance of self-management activities, improve patient lifestyle behaviours, and patient self-efficacy in making behaviour changes. These changes were associated with improved perception of quality of life.

Becker et al. [30] compared outcomes of three groups of patients with low back pain from one control group of several practices and two intervention groups. The control group comprised of 43 practices with 410 patients and received printed educational information alone. One intervention group received education on guideline implementation (GI) alone (37 practices, 479 patients) and the other intervention group received GI plus training in motivational interviewing techniques (MIT) (38 practices, 70 practice nurses, 489 patients). Both intervention group patients showed significant improvements in functional capacity at six months though there was no difference at 12 months. Patients of the clinicians in the motivational interviewing group showed significant improvement in functional capacity after six months with a greater number of patients reporting pain free days (less permanent pain) after six months and after 12 months. These same patients also demonstrated a significant improvement in quality of life scale, having received one session from a practice nurse trained in motivational interviewing. The control group demonstrated no improvement in functional capacity, no perceived improvement in quality of life and they reported greater levels of permanent pain compared to both intervention groups.

Rubak et al. [31] included 65 GPs from 48 practices and all GPs attended a half day course on intensive treatment of type-2 diabetes. Practices were then randomised into control and intervention groups and the intervention group had an additional one and a half day residential course for 29 GPs from 21 practices (137 patients) on motivational interviewing techniques. The intervention practices also had a half day follow up course on MIT, twice during the following one year. The patients of the intervention group clinicians were shown to be more motivated to change behaviours and significantly more autonomous in their choice of actions leading to behavioural changes than patients of the control clinicians. The patients of the intervention clinicians also reported having received significantly more specific advice from their GP regarding diet, exercise and self-control of diabetes, and they also reported receiving significantly more counselling re smoking cessation. Intervention group patients reported an increase in their awareness of the importance of taking control of their own specific risk factors for diabetes. This study also reported a significant improvement in self-efficacy between pre-training and post training for all primary care health professionals undergoing motivational interviewing training.

## Discussion

### Main findings

The key finding of this systematic review is the scarcity of studies that assess the impact on patient outcomes of training primary care clinicians in patient self-management of chronic conditions. This was surprising given that patient self-management is a core element of person-centered healthcare in family practice and given the volume of published material on patient self-management.

This review shows that when health professionals undergo training in empowering patients for self-management of chronic conditions, it is possible to achieve improvement in patients’ self-efficacy, autonomy and motivation to change, functional capacity, pain free days and quality of life.

One study [30] demonstrated improvement in functional capacity, quality of life scale and a greater number of pain free days reported by patients after six months and after 12 months among those whose primary care clinicians had been given training in motivational interviewing techniques. A second study [31] showed that patients of the health professionals who participated in specific training programmes were more motivated to change behaviours and were more autonomous in their choice of behavioural changes compared to a control group. These patients were significantly more aware of the importance of controlling their diabetes for specific factors, and had a higher level of perception of having received specific advice from their GP on healthy behaviour changes.

### Limitations of the review

Despite much literature on patient self-management in chronic disease, focussing on whether training health professionals regarding patient self-management improves patient outcomes, resulted in only two articles being eligible for inclusion in this systematic review. We are aware that some additional studies have been published more recently on self-management that were outside the chosen search period of this review. We plan to update this review in 2018. However, we feel it is important to publish the findings of this first phase review to highlight that, despite a vast volume of literature on one topic, evidence of impact on patient outcomes is largely lacking during the study period despite the publicity and interest in patient empowerment for self-management in the years leading up to its official inclusion in the European definition in 2011.

The small number of studies included and the range of outcome measures therein made concrete conclusions impossible, both papers describe positive outcomes from teaching motivational interviewing skills to clinicians, but we do not yet know if other approaches would be equally or more effective. The two studies did not report effect sizes, further complicating the interpretation of results. A further limitation is that only articles in English were included, based on available resources.

A total of 1643 patients, 191 clinicians and 164 practices were involved in these studies in two European countries, however further research on this topic is also needed to clarify if other factors are effective in improving patient outcomes other than those involving time constrained clinicians in general practice.

### Interpretation of findings in the context of existing evidence

This review has given us concrete evidence of the lack of studies in the English language on improving patient outcomes through training primary care clinicians in patient self-management. Previous studies focus on patient education, group discussion among patients, shared experiences and unstructured acquisition of knowledge during clinical encounters or through leaflets and brochures [16, 25, 32, 33] rather than on assessing the specific effect of specific clinician training on patient outcomes in this setting. Primary care professionals have a longitudinal relationship with patients in the patient’s own community, are accessible to patients, and though contacts are intermittent, there is coordination and continuity of care. This review suggests that teaching motivational interviewing skills to health professionals in primary care may improve self-efficacy and quality of life in their patients, compared to those patients of clinicians who did not participate in this training. It suggests that the addition of motivational interviewing techniques to usual care may have added benefit for patients over usual care in the primary care setting, however further research is needed to identify if other educational interventions or skills are useful.

### Implications for further research

Further research is needed to distil the specific techniques to empower patients for self-management [34]; to explore and define the various aspects of the concept of patient empowerment [35]; and the variety of approaches that can be taken by primary care physicians to support patients to self monitor and make decisions about their chronic condition. We also need to identify and address potential barriers for self-management in patients [36].

Patients with chronic conditions interact over time with many professionals in primary and secondary care. There are many other interventions that may help to improve patient self-management, (for example group education, health coaching, telemedicine, e-health, media led interventions, voluntary associations, sports organisations and community group activities). Additional factors having a potential impact on successful outcomes include patient preferences for individual or group interventions, and patients’ values, goals, level of education and literacy. Patients have increasingly easy and direct access to online resources. We need research to guide both clinicians and patients to know which methods are best used in which settings [37], and which methods are not suited to particular settings. Careful designs and methods need to be used in future studies to assess the impact of such factors on measuring the outcomes of patient self-management including the Hawthorne effect and language bias. Appropriate assessment tools are required taking account of the integrative model of change appropriate in different settings [38].

Studies are also needed on evidence for the feasibility of training health professionals in patient self-management approaches including efficient use of resources such as time, people and finance, as research into the value and effectiveness of the various methods that can be used to “empower patients” is still in its infancy [37].

We also need to promote the inclusion of validated scales and instruments in future research for measurement and comparison of patient outcomes in chronic conditions.

## Conclusions

Effective training for healthcare staff in patient self-management support is important in the context of patient centred care, patient outcomes, health care economics, strategy and delivery of healthcare on a global perspective.

This review suggests that primary care health professionals can help to harness patients’ capacity to contribute to improvement of their own health outcomes. Despite increasing literature on patient self-management and on health professional training on this topic, the evidence is very limited on measured patient health outcomes up to two years after patient self-management was incorporated into the WONCA definition of general practice. We plan to undertake a follow-up systematic review to establish if this changes over time or if further research is needed to assure health professionals and policy makers that patient self-management is a worthwhile and effective aspect of general practice.

## Additional file

Additional file 1: Search terms and search example. (DOCX 15 kb)

## Abbreviations

CCM: Chronic care model
EQUIP: European Society for Quality and Safety in General Practice / Family Medicine
GI: Guideline implementation
GP/FM: General Practitioner/ Family Physician
MIT: Motivational interviewing techniques
NCD: Non-communicable disease
PSM: Patient self-management
RCT: Randomised controlled trial
WHO: World Health Organisation
WONCA: World Organization of National Colleges, Academies and Academic Associations of General Practitioners/Family Physicians
