# Supplementary material for: Does patient self-management education of primary care professionals improve patient outcomes: a systematic review
Source: BMC Fam Pract. 2018 Sep 29;19:163. doi: 10.1186/s12875-018-0847-x (PMC6164169; doi:10.1186/s12875-018-0847-x)
Supplement: Supplementary file 1 — Search terms and search example. (DOCX 15 kb) [file 12875_2018_847_MOESM1_ESM.docx]

**Additional Material – Search Terms and Search Example**

The search terms in the search strategy were:

1. primary healthcare
2. physicians, primary care
3. physicians, family
4. general practitioners
5. dental
6. pharma*
7. #1 OR #2 OR #3 OR #4 NOT #5 NOT #6
8. education, continuing
9. evidence-based medicine/education
10. patient education as topic
11. training
12. #8 OR #9 OR #10 OR #11
13. “long-term care”
14. chronic disease
15. #13 OR #14
16. #7 AND #12 AND #15

**Search example as conducted in PubMed:**

**History**

[Clear history](http://www.ncbi.nlm.nih.gov/pubmed/advanced)

| Recent queries | | | | |
| --- | --- | --- | --- | --- |
| **Search** | **Add to builder** | **Query** | **Items found** | **Time** |
| [#17](http://www.ncbi.nlm.nih.gov/pubmed/advanced) | [Add](http://www.ncbi.nlm.nih.gov/pubmed/advanced) | Search **(#3) AND #16** | [610](http://www.ncbi.nlm.nih.gov/pubmed/?cmd=HistorySearch&querykey=17) | 05:48:37 |
| [#16](http://www.ncbi.nlm.nih.gov/pubmed/advanced) | [Add](http://www.ncbi.nlm.nih.gov/pubmed/advanced) | Search **("long-term care") OR "chronic disease"** | [248183](http://www.ncbi.nlm.nih.gov/pubmed/?cmd=HistorySearch&querykey=16) | 05:48:13 |
| [#15](http://www.ncbi.nlm.nih.gov/pubmed/advanced) | [Add](http://www.ncbi.nlm.nih.gov/pubmed/advanced) | Search **("long-term care") OR chronic disease** | [477921](http://www.ncbi.nlm.nih.gov/pubmed/?cmd=HistorySearch&querykey=15) | 05:46:59 |
| [#14](http://www.ncbi.nlm.nih.gov/pubmed/advanced) | [Add](http://www.ncbi.nlm.nih.gov/pubmed/advanced) | Search **((#3) AND "long-term care") OR chronic disease** | [452690](http://www.ncbi.nlm.nih.gov/pubmed/?cmd=HistorySearch&querykey=14) | 05:46:22 |
| [#13](http://www.ncbi.nlm.nih.gov/pubmed/advanced) | [Add](http://www.ncbi.nlm.nih.gov/pubmed/advanced) | Search **((#3) AND long-term care) OR chronic disease** | [453070](http://www.ncbi.nlm.nih.gov/pubmed/?cmd=HistorySearch&querykey=13) | 05:45:21 |
| [#12](http://www.ncbi.nlm.nih.gov/pubmed/advanced) | [Add](http://www.ncbi.nlm.nih.gov/pubmed/advanced) | Search **(#3) AND long-term care** | [544](http://www.ncbi.nlm.nih.gov/pubmed/?cmd=HistorySearch&querykey=12) | 05:44:50 |
| [#10](http://www.ncbi.nlm.nih.gov/pubmed/advanced) | [Add](http://www.ncbi.nlm.nih.gov/pubmed/advanced) | Search **(#9) AND chronic disease** | [19](http://www.ncbi.nlm.nih.gov/pubmed/?cmd=HistorySearch&querykey=10) | 05:38:12 |
| [#9](http://www.ncbi.nlm.nih.gov/pubmed/advanced) | [Add](http://www.ncbi.nlm.nih.gov/pubmed/advanced) | Search **(#3) AND patient empowerment** | [153](http://www.ncbi.nlm.nih.gov/pubmed/?cmd=HistorySearch&querykey=9) | 05:37:46 |
| [#7](http://www.ncbi.nlm.nih.gov/pubmed/advanced) | [Add](http://www.ncbi.nlm.nih.gov/pubmed/advanced) | Search **(#4) AND patient participation** | [84](http://www.ncbi.nlm.nih.gov/pubmed/?cmd=HistorySearch&querykey=7) | 05:35:42 |
| [#6](http://www.ncbi.nlm.nih.gov/pubmed/advanced) | [Add](http://www.ncbi.nlm.nih.gov/pubmed/advanced) | Search **(#3) AND patient participation** | [800](http://www.ncbi.nlm.nih.gov/pubmed/?cmd=HistorySearch&querykey=6) | 05:34:59 |
| [#4](http://www.ncbi.nlm.nih.gov/pubmed/advanced) | [Add](http://www.ncbi.nlm.nih.gov/pubmed/advanced) | Search **(chronic disease) AND #3** | [871](http://www.ncbi.nlm.nih.gov/pubmed/?cmd=HistorySearch&querykey=4) | 05:32:58 |
| [#3](http://www.ncbi.nlm.nih.gov/pubmed/advanced) | [Add](http://www.ncbi.nlm.nih.gov/pubmed/advanced) | Search **(#1) AND #2** | [12549](http://www.ncbi.nlm.nih.gov/pubmed/?cmd=HistorySearch&querykey=3) | 05:32:16 |
| [#2](http://www.ncbi.nlm.nih.gov/pubmed/advanced) | [Add](http://www.ncbi.nlm.nih.gov/pubmed/advanced) | Search **((education, continuing) OR evidence-based medicine/education) OR patient education as topic** | [131718](http://www.ncbi.nlm.nih.gov/pubmed/?cmd=HistorySearch&querykey=2) | 05:31:49 |
| [#1](http://www.ncbi.nlm.nih.gov/pubmed/advanced) | [Add](http://www.ncbi.nlm.nih.gov/pubmed/advanced) | Search **primary healthcare OR physicians, primary care OR physicians, family OR general practitioners NOT dental NOT pharma*** | [189645](http://www.ncbi.nlm.nih.gov/pubmed/?cmd=HistorySearch&querykey=1) | 05:28:28 |
